# A Lecture on Gun Shot Wounds

**Published:** 1852-10

**Authors:** 


*A Lecture on Gun Shot Wounds.* By Richard McSherry, M. D.

This is an excellent lecture, and upon a subject, too, in which every free American has a personal interest, as he does not know how soon some other free American may shoot him. Pistols are now carried as toothpicks used to be. We suggest, that in view of the state of things in our city, that this lecture be delivered to the public schools.

